# Drought-Adapted Mediterranean Diet Plants: A Source of Bioactive Molecules Able to Give Nutrigenomic Effects per sè or to Obtain Functional Foods

**DOI:** 10.3390/ijms25042235

**Published:** 2024-02-13

**Authors:** Silvia La Scala, Flores Naselli, Paola Quatrini, Giuseppe Gallo, Fabio Caradonna

**Affiliations:** 1 Dipartimento di Scienze e Tecnologie Biologiche Chimiche e Farmaceutiche, Sezione di Biologia Cellulare, Università di Palermo, 90128, Palermo, Italy; silvia.lascala@unipa.it (S.L.S.); paola.quatrini@unipa.it (P.Q.); giuseppe.gallo@unipa.it (G.G.); fabio.caradonna@unipa.it (F.C.); 2 NBFC—National Biodiversity Future Center, 90133 Palermo, Italy

**Keywords:** plant-derived compounds, nutrigenomics, functional food, biofortified food, environmental factors, healthy diet, microbiome

## Abstract

The Mediterranean diet features plant-based foods renowned for their health benefits derived from bioactive compounds. This review aims to provide an overview of the bioactive molecules present in some representative Mediterranean diet plants, examining their human nutrigenomic effects and health benefits as well as the environmental advantages and sustainability derived from their cultivation. Additionally, it explores the facilitation of producing fortified foods aided by soil and plant microbiota properties. Well-studied examples, such as extra virgin olive oil and citrus fruits, have demonstrated significant health advantages, including anti-cancer, anti-inflammatory, and neuroprotective effects. Other less renowned plants are presented in the scientific literature with their beneficial traits on human health highlighted. Prickly pear’s indicaxanthin exhibits antioxidant properties and potential anticancer traits, while capers kaempferol and quercetin support cardiovascular health and prevent cancer. Oregano and thyme, containing terpenoids like carvacrol and γ-terpinene, exhibit antimicrobial effects. Besides their nutrigenomic effects, these plants thrive in arid environments, offering benefits associated with their cultivation. Their microbiota, particularly Plant Growth Promoting (PGP) microorganisms, enhance plant growth and stress tolerance, offering biotechnological opportunities for sustainable agriculture. In conclusion, leveraging plant microbiota could revolutionize agricultural practices and increase sustainability as climate change threatens biodiversity. These edible plant species may have crucial importance, not only as healthy products but also for increasing the sustainability of agricultural systems.

## 1. Introduction

Italy is one of the richest European countries in terms of plant species: in fact, this country has about half of the recognized plant species in Europe, including edible plants, which grow wild thanks to the mild climate and soil fertility. From a nutritional point of view, the healthful properties of Mediterranean species are due to a perfect balance of essential nutrients, such as vitamins and minerals, and the presence of beneficial bioactive compounds [1].

As reported in different reviews specifically focusing on their ecological, phylogenic, and evolutionary characteristics [2,3,4], Mediterranean plants generally consist of a complex mixture of taxa having various biogeographical origins and evolutionary histories, with approximately 50% of them considered endemic. Although very diverse, Mediterranean plants are stress-tolerant species, including evergreen trees and shrubs, semi-deciduous shrubs, geophytes, and winter annual herbs. These plants share morphological, anatomical, and phenological traits according to an evolutionary convergence driven by environmental conditions, such as climate. 

In particular, we focused our attention on some wild and spontaneous Mediterranean edible plants, which represent a great resource, fitting fully into nutritional plans as ingredients in many traditional culinary recipes. In particular, this review addresses two Mediterranean aromatic herbs (*Thymus vulgaris*. and *Origanum vulgare*) endowed with extraordinary antioxidants, antibacterial, anti-inflammatory properties [5], and at the same time, tasty ingredients for our cuisine, and two xerophilous cultivated and naturalized drought-resistant shrubs, *Opuntia ficus indica* and *Capparis spinosa*, of promising potentialities to cope with emerging climatic change in the Mediterranean countries. This manuscript focuses on some products from the plant’s secondary metabolism, a group of reactions that the plant uses to interrelate with the external environment. The reaction products are the secondary metabolites, of which bioactive compounds like phenols, terpenes, and alkaloids are part.

These edible wild plants are rich in many metabolites that are often referred to as secondary since they are generally not essential for the growth of the producer organism (at least under controlled conditions) and are produced in smaller quantities than the primary metabolites. However, they play a key role in the interactions between plant organisms and the biotic and abiotic environment in which plants live. In particular, it is known that secondary metabolites act as protection factors against microbial pathogens [6], facilitate reproductive processes, and act as defense mechanisms against abiotic stresses [7]. It has become increasingly clear that these compounds are involved in numerous biochemical and physiological processes and that they can play a role as protective health factors in improving plant growth and development. 

In addition, polyphenols, carotenoids, and terpenes have been shown in various experimental studies to exert preventive action against chronic degenerative diseases in human beings [8] thanks to their antioxidant activity, which is mainly expressed in opposing oxidative processes. 

The need, therefore, came about to draw up a diet that included these plants. The precious contribution they give to a healthy diet is schematically shown in an Eating Pyramid presented by Mantzioris and Villani [9], indicative of the purposes of complete and balanced nutrition. The pyramid is divided on the size of the sector, which indicates the frequency of intake/relative quantity ratio for each specific food depicted. According to the information presented, it can certainly be said that no restrictions are imposed regarding fruits, to which the prickly pear belongs, and spices, such as oregano and rosemary, the consumption of which is free from any restrictions. Thus, a balanced mix of healthy nutrients and benefits for human health constitutes what we commonly call the “Mediterranean diet”, a UNESCO World Heritage Site since 2010. Numerous studies link it to longevity and protective effects against several diseases, including a nutritional-epigenetic effect on cancer cells [10,11,12].

In order to provide an insight into some of the best-known flavonoids and terpenoids that can be extracted from Mediterranean plants, a precise bibliographic choice has been made as functional for the purpose of the review. Four xerophilous Mediterranean species that naturally inhabit semi-arid environments and are rich in bioactive molecules have been selected as models of economic crops that can fulfill both environmental and health purposes.

Indeed, except for an essential part of historical articles peculiar to some concepts, most of the selected papers (over 70%) are no older than 2014. The results highlighted a widespread scientific interest in flavonoids and terpenoids mainly produced from edible Mediterranean plants, whose major characteristics are described and discussed below. 

## 2. Mediterranean Edible Plant Bioactive Molecules: An Arsenal of Pigments, Flavonoids, and Terpenoids

Bioactive molecules are naturally contained in a huge number of foods, mostly plants, and are grouped into classes based on structural or biosynthetic characteristics. They can improve certain functions of the organism, which is why they are suggesting more interest in the scientific community, and can positively influence a physiological benefit and/or reduce the risk of developing certain diseases [13,14]. Secondary metabolites, which bioactive molecules belong to, are not essential for the development of the plant or the reproduction of the organism but represent useful products for beneficial purposes [15,16] or are involved in protective functions against infections [17,18] or physiological stresses like UV radiation [19]. Most plants have not yet been examined for secondary metabolites, and new compounds are being discovered day by day. It can, therefore, be said that bioactive molecules are still a rich but unexplored world that gains charm when generic bioactivity can be explicated in power to modulate gene expression in cells. The purpose of the following sub-paragraphs is to inform about the structure, activity, and application potential of determined biomolecules contained in prominent Mediterranean edible plant species growing on semi-arid soils, as well as investigate their healthy properties.

### 2.1. Indicaxanthin: The Yellow Pigment of Prickly Pear Fruit

The prickly pear, or *Opuntia ficus-indica L. Mill*, is an iconic symbol of Southern Italy and holds within it a treasure of nutrients [20]. *O. ficus-indica L. Mill* (order Caryophyllales family Cactaceae) is native to Mexico but naturalized and widely cultivated in Mediterranean countries. Plants of this genus prefer hot and dry climates, which is why Mediterranean countries are an ideal habitat for their growth, to such an extent that they have been considered invasive in Sicily. There are many varieties of the prickly pear: it is multicolored, and the pulp can be orange, green, white, yellow, or red. The outward appearance, besides being a well-studied Physico-chemical phenomenon described above, provides a considerable quality parameter of the products. Hence, the consumer is naturally inclined to choose a more colorful product as it is more appealing. Prickly pear extracts contain a large amount of these biomolecules, which is why they have always been tested for their antioxidant capabilities, yielding promising results [21,22]. Moreover, fruits and their juices have always been recommended for their diuretic, hypoglycemic, analgesic, and anti-inflammatory effects, as well as for gastritis relief [20]. Regarding the use of the isolated molecules, there are extensive studies on betanin, which is shown to have great antioxidant activity in inhibiting lipid peroxidation of membranes [23]. Among the phytochemicals, indicaxanthin has attracted the scientific community’s attention. The molecule belongs to the family of betaxanthin of the betalain class: vacuolar pigments composed of a central nitrogenous structure, betalamic acid [24]. Betalamic acid condenses with imine compounds to form betacyanins or betaxanthins. More specifically, indicaxanthin derives from the condensation of betaine with L-proline [25]. Since it belongs to the plant pigment class, it is a light-adsorbing unit and must, therefore, be preserved from exposure to direct light to avoid damage to the structure. It is also susceptible to enzymatic reactions and temperature fluctuations. The same is said of Indicaxanthin, where we note its properties as a redox agent [26], as a protector against hemolysis [27], and as a pigment capable of crossing the blood-brain barrier when administered in nutritionally relevant quantities [28]. Recent studies have also shown the role of indicaxanthin as a modulator of DNA methylation in Caco2 cells, making this molecule a good candidate for an anticancer drug [12], and its pro-autophagic potential in human colorectal cancer cells [29].

### 2.2. Kaempferol and Quercetin: Flavonoids in Caper Plant 

**Figure 1:**
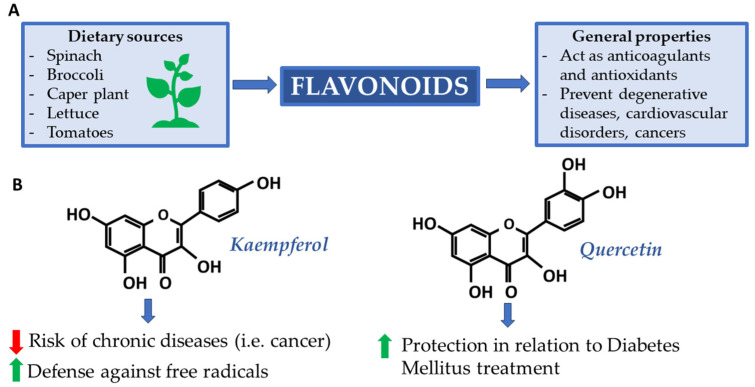
(**A**) Supply sources and main flavonoid properties; (**B**) Kaempferol and quercetin: two flavonoids renowned for their beneficial properties.

Flavonoids are a large class of phenolic compounds. They can exert a variety of beneficial biological activities and have proven to be fundamental for health, especially in the prevention of degenerative diseases, cardiovascular disorders, and cancers [30]. From a chemical point of view, most flavonoids consist of a core structure composed of 15 carbon atoms (C-15) distributed over three rings: two benzyls and one heterocyclic. Kaempferol (C_15_H_10_O_6_) and quercetin (C_15_H_10_O_7_) are two flavonols with very similar structures, as can be seen in Figure 1. 

Preliminary studies have shown that crude extracts containing flavonoids (including quercetin and kaempferol derivatives) of certain plants have anticoagulant and antioxidant properties in human plasma [31]. It is crucial to control oxidation reactions because, although they are physiological, they can produce excess free radicals. These, when overexpressed, are responsible for cellular damage. In this, antioxidants play a crucial role, acting as reducing agents and stopping these reactions [32]. The caper plant (Capparis spinosa L. order Brassicales, family Capparaceae), widespread throughout the Mediterranean basin, is rich in quercetin and kaempferol. As reported by the USDA Database for the Flavonoid Content of Selected Foods [33], the caper plant classifies among the best plant species in terms of quercetin and kaempferol content by total weight. This makes it an ideal candidate for future studies and application, especially in Type 2 Diabetes Mellitus treatment (T2DM). Some interesting results demonstrate, in fact, that quercetin, among all, interacts with DNA and shows protective effects in relation to T2DM [34,35]. In 2020, Anachuna et al. investigated the nutrigenomic effects of quercetin and kaempferol concerning their impact on prenatal and postnatal food deprivation-induced developmental anomalies in rats. Both compounds demonstrated significant effects in mitigating the alterations induced by these food restrictions [36]. These findings suggest that quercetin and kaempferol have nutritional genomic properties, which would counteract the negative effects of prenatal and postnatal malnutrition on pregnancy outcomes and developmental trajectories. The nutrigenomic effects of quercetin and kaempferol have also been observed in studies focusing on cancer pathways [37]. These flavonoids exert their effects by affecting gene expression, particularly concerning DNA methylation and histone acetylation, and have been shown to modulate genes related to cell growth regulation and apoptosis in other mechanisms of cancer progression [37,38]. Moreover, these agents increase genomic stability, inhibit VEGF signaling, and have been reported to have antiviral activity [39]. In addition, these flavonoids have shown promise in inhibiting histone deacetylases (HDACs) that affect histone acetylation and gene expression [40]. This inhibition leads to increased histone acetylation, downregulation of specific genes, and cell cycle arrest/induction of apoptosis. In summary, quercetin and kaempferol, present also in the caper plant, exhibit nutrigenomic effects by influencing gene expression through mechanisms like DNA methylation regulation, histone acetylation inhibition, and promoting genomic stability. The epigenetic effects of these flavonoids selectively target cancer cells while inhibiting normal cell proliferation.

### 2.3. Carvacrol and γ-Terpinene: Antimicrobial Terpenoids in Oregano and Thymus 

Aromatic spices such as oregano and thyme are used every day to flavor dishes of the Mediterranean diet. Both aromatic herbs, *Thymus* spp. and *Origanum* spp., belong to the order Lamiales in the family Lamiaceae. They are well known for their antioxidant effects when appropriately accompanied by cooked dishes [41,42]. 

**Figure 2:**
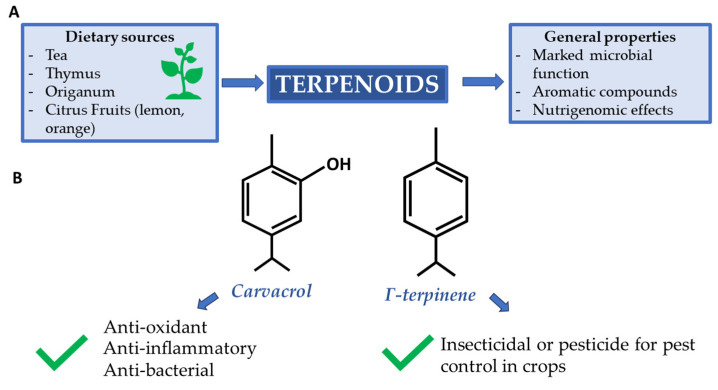
(**A**) Supply sources and main terpenoid properties; (**B**) Carvacrol and γ-terpinene are two terpenoids renowned for their beneficial properties.

Carvacrol and γ-terpinene, whose structures are shown in Figure 2, are two terpenoids that can be produced by the above-mentioned species. Carvacrol is one of the components in thyme and oregano essential oil with the highest microbial activity. It is known for its ability to inhibit flagellum development in *Escherichia coli* and to trigger heat shock proteins during infections [43,44]. 

Γ-terpinene has a similar function to carvacrol. Unlike the former, it must be inoculated with other terpenoids in bactericidal assays to have an appreciable effect [45]. Possibly, its reduced activity, compared to carvacrol, is due to the less detectable amount in the extracted essential oils.

Carvacrol has also been demonstrated to have nutrigenomic action in changing the host ileum microbial population dynamics to increase the abundance of some healthy bacterial species in the chicken gut [46].

Reference should be made to the class of terpenoids: the oxygenated derivatives of terpenes. These are hydrocarbons consisting of one or more isoprene units, depending on whether they are monoterpenes, sesquiterpenes, diterpenes, polyterpenes, etc. The aforementioned class of compounds is widely represented in both the plant and animal worlds [47,48]. In particular, monoterpenes, sesquiterpenes, and diterpenes are abundant in the essential oils of plants and have a marked antimicrobial function [49,50] and give each plant a characteristic odor or aroma. D-Limonene, among all, is a major constituent of Citrus essential oils, also used as a cancer chemotherapeutic compound; it shows its principal nutrigenomic effect on dividing cells, preventing assembly of mitotic spindle microtubules affecting chromosome segregation and cytokinesis, thus causing aneuploidy and genomic instability [51].

## 3. Mediterranean Functional Food and Biofortified Products 

Mediterranean plants are a rich source of secondary metabolism products, in particular phenols, terpenes, and alkaloids [52]. They provide a model for the design of the so-called "Biofortified Foods," which are enriched foods supplemented with bioactive molecules (antioxidants, fiber, and omega-3 fatty acids), of which plants are active producers.

Functional foods are described as foods that positively affect one or more physiological functions. A fundamental prerogative of these foods is to help preserve or improve health status and/or reduce the risk of occurrence of diet-related diseases. Mediterranean diet, already known for its numerous beneficial properties, is rich in these functional foods since bioactive components are naturally present in the plant species under discussion [53]. These foods, although they differ from each other, share a common goal: to provide nutritious, healthy benefits. 

Fortified foods, on the other hand, although they bring beneficial properties to human health, are those that are enriched with specific nutrients that are deficient in the diet. The enrichment is made through conventional cultural techniques or through genetic modifications aimed at increasing essential nutrients (vitamins, minerals, and amino acids) in crops [54].

The Mediterranean diet, renowned for its health benefits, offers a rich source of functional and/or biofortified foods. The health effects of these functional foods have been investigated in many scientific studies. A wide variety of fortified foods is rich in bioactive metabolites such as phytochemicals, antioxidants, fibers, probiotics, or omega-3 fatty acids. Indeed, these substances can help prevent various chronic diseases such as cardiovascular problems, diabetes, and cancer.

An example is a study by Visioli and colleagues (1998) that highlighted the cardiovascular benefits of consuming dietary fats such as extra virgin olive oil [55]. The current literature counts many studies that have shown that phenolic compounds in olive oil (in particular extra virgin olive oil: EVOO) are bioactive molecules with anti-cancer, anti-inflammatory, anti-aging, and neuroprotective properties, and its ability to induce changes in DNA methylation patterns have been highlighted [56]. It must also be considered that it is able to genetically modulate cellular pathways related to oxidative mechanisms. A review was proposed by Serreli 2020 [57] highlighting the existing literature regarding the interaction between EVOO polyphenols and the NF-κB and Nrf-2 signaling pathways, two important modulators of age-related disorders and aging.

Polyphenols can induce changes in chromatin structure by interacting with DNA methyltransferases and histone-modifying enzymes, affecting gene expression associated with inflammation, oxidative stress, and cellular metabolism [16,58]. 

In addition, a study conducted by Sesso and colleagues (2003) examined the association between the consumption of lycopene, contained primarily in tomatoes, and the reduced risk of cardiovascular disease (CVD) [59]. 

Another notable example includes the work of Ghanim and colleagues (2011), who have related orange juice intake to a high-fat, high-carbohydrate meal (HFHC), demonstrating antioxidant and anti-inflammatory effects of citrus flavonoids in attenuating metabolic complications related to the syndrome [60]. These observations may help to explain the mechanisms underlying oxidative stress and postprandial inflammation, the pathogenesis of both insulin resistance and atherosclerosis. 

On the other hand, biofortified Mediterranean foods are intentionally improved to increase their nutritional contents. Among these, wheat, like many other staple grains, contains low levels of essential micronutrients: iron and zinc. Filippa Borrill’s (2014) [61] review discusses some approaches to improve the content of the two aforementioned micronutrients, using the existing knowledge on model herbs. 

The inclusion of these kinds of products in daily consumption would be a successful strategy to fight malnutrition, which is particularly widespread in poorly industrialized countries. Promising effects have been observed when biofortified cereals such as zinc-fortified wheat or iron-fortified rice are given to patients affected by anemia. In particular, they exert an effect on the metabolic pathways and reduce the risk of nutrition-related diseases [62]. In addition, supplementation of biofortified foods not only provides numerous nutritional benefits but also contributes to a slower occurrence of non-communicable diseases (NCDs) such as diabetes, obesity, and neurodegenerative diseases. Studies by Gomez-Galera et al. (2010) highlight the positive impacts of consuming biofortified crops, in particular rice and wheat, on improving cognitive function and reducing the risk of age-related cognitive decline. [63] It also highlights the positive impacts of consuming biofortified crops, particularly rice and wheat, on improving cognitive function and reducing the risk of age-related cognitive decline.

## 4. Drought-Resistant Plants in the Mediterranean Diet: An Opportunity to Mitigate the Effects of Climate Change

Mediterranean diet plants are known for their ability to adapt to aridity. Many varieties in the area must implement different strategies to survive when water is short: one of the best known is "drought escape," a strategy implemented by annual plants to ensure growth and reproduction. With climate changes, the plants that inhabit the Mediterranean brush are constantly subjected to new challenges and, therefore, must prove not only resilient but also equally productive in order to preserve the varieties’ survival. Some species, particularly shrubs to which aromatic plants belong, have a protective role for the soil; in fact, they are used in areal cover to screen it from raindrops and as protection for erosion [64,65].

Soil is involved in physicochemical phenomena that depend on climate and interaction with living organisms. The environmental aspects influence soil fertility, acting on the productive potential of the cultivated and wild plants. As observed in several studies, all this results in changes in the level of primary and secondary metabolism. Mehalaine S. and colleagues (2020) studied the effect of soil properties on the accumulation of essential oils in two spontaneous plants growing in the Algerian semi-arid region, thyme and rosemary, and noted that pH and nitrogen:phosphorus ratio (N:P) had a positive correlation with the concentration of essential oils in both species. The two elements, nitrogen and phosphorus, exert their synergistic effect on the assimilation of primary metabolites and, consequently, of secondary metabolites [66].

In addition to having beneficial effects on plant growth and health, the microbiota can influence plant metabolites qualitatively and/or quantitatively. Some bacteria, the already known Plant Growth Promoting Bacteria (PGPB), can produce hormones, provide siderophores to the host plant, trigger a cross-talk modulating the production of phytohormones, or even increase the tolerance of the plant to reported stress (for example, drought). It then triggers a mutualism in which the plant produces the initial unit of the biosynthetic pathways, and the endophytes complete the remaining biosynthetic phases until the final compound [67]. 

The species belonging to the Mediterranean area are interesting not only from a nutritional point of view but also from an ecological and adaptive point of view since they grow well in arid environments. In a period where rising temperatures are changing weather models and threatening biodiversity, the need to find strong, adaptable species such as aromatics and shrubs is becoming more and more concrete. Various attempts are made to exploit the available sources to better face this problem, especially on the agro-alimentary front, where increasingly eco-sustainable approaches are preferred to optimize the available resources as much as possible [68]. Now more than ever, it is fundamental to focus on those autochthonous plants adapted to arid climates. These plants survive in climates where water shortages are frequent, and this is going to be more and more frequent in the Mediterranean regions where drought periods are becoming longer, and rains are heavier and reduced to shorter periods throughout the year. Many selective factors contribute to increasing the yield of plants in these harsh conditions, and the soil microbiota plays an increasingly decisive role [69]. Sicily, a southern region of Italy located at the center of the Mediterranean basin, is one of the regions more affected by desertification and, at the same time, a hotspot of plant diversity and the home of many plants resistant to drought and adapted to high temperatures.

Among them, *O. Ficus indica L. Mill* is one of the most widespread wild and cultivated drought-resistant species rich in bioactive molecules, whose fruits (prickly pears) are known for both their pleasant taste and beneficial properties, especially in the nutrigenomic/epigenetics field [70,71,72]. 

Beyond *Opuntia*, spices and aromatic herbs are important products since they have been cultivated and used in everyday life for millennia [73]. Especially in Sicily, they have been fundamental in the evolution of recipes and traditional cuisine. The most common species include oregano (*Origanum vulgare*), thyme (*Thymus vulgaris*), rosemary (*Rosmarinus officinalis*), mainly used to flavor meat and side dishes; and the caper plant (*Capparis spinosa*) whose buds and fruits are added to salads. All these xerophilous species are considered multi-value and promising crops with high potentialities for agrosystems under the threat of global warming [74].

Therefore, these species combine two key aspects: being particularly beneficial from a nutritional and salutary point of view and being optimal candidates for the valorization and protection of soils under desertification risk.

## 5. Mediterranean Diet Plant Microbiome: A Need, a Source, a Perspective for the One Health Approach

Interactions between plants and soil microorganisms are most of the time beneficial to their growth and adaptation. Several studies have shown that when interactions are established, both living species take part in this collaboration: plants improve nutrient assimilation and tolerance to abiotic and biotic stresses, while microbiota members take advantage of the presence of organic compounds produced by plants [75].

With the advancement of genomic technologies, it is possible to characterize both the soil and plant metagenomes, which provides detailed information about the resident microbial population. In this regard, the most used approach is metataxonomics which analyses metagenomic DNA content to infer microbiota structures differing according to plant species, soil chemical physical variables, soil cover, and climate [76,77].

In the rhizosphere, particularly favorable conditions are created for the life of numerous plant-useful microorganisms, such as the most studied soil bacteria called rhizobia. They settle in the roots of legume hosts, inducing the formation of typical root nodules, and here, they fix nitrogen while using organic compounds and mineral salts from the host. Meanwhile, they produce nitrogen-assimilable compounds, which are exploited as a source by the plant. Many different bacterial species known as plant growth promoters (PGP) favor plant’s rooting and boost the uptake of macroelements (N, P, K) and microelements present in the soil; beyond bacteria, mycorrhizal fungi, are essential to more than 80% plant species. These micro-organisms perform their specific action, which is made available to the plant and exploited massively thanks to the large root system. The best results for crops are obtained from the ternary association of roots-mycorrhizae-useful micro-organisms [78,79].

Plant-associated microorganisms are also often classified on the type of association they establish: endophytic when they thrive in the plant tissues of hypogeal (i.e., roots) or epigeal (i.e., shoots, leaves) organs of a plant [80]; symbiotic each time they establish a close and long-term interaction with a biological organism. Symbiotic interaction confers an advantage for both bacteria and plants since the former uses nutrients for their growth, and the latter influences the enrichment of the soil microbiome. Many studies investigate this topic: one, in particular, focuses on how bacteria establish symbiotic relationships with Vetiver roots, wild grass whose essential oil is fragrant and widely used in perfumery [81], and another one puts the attention on the role of endophytes in enhancing the production of bioactive secondary metabolites The host-specific secondary metabolites produced by endophytes, their therapeutic properties and host-endophytes interaction with production of bioactive secondary metabolites and the role of endophytes in enhancing the production of bioactive secondary metabolites is discussed [82].

The microbial diversity of Mediterranean plants has been observed and analyzed through the years, and although there are still not many studies to support it, a basic microbiological identity can still be defined for most of the plant species considered. Table 1 lists some of the most abundant bacterial taxa found in the Mediterranean plants considered in this minireview, divided into the plant compartments analyzed. 

The microbiota thus constitutes a source of biodiversity and, at the same time, represents a biotechnological opportunity to develop alternative agricultural methods since it exerts important effects on growth promotion in plants [88]. All good practices to enhance and safeguard the soil microbiota in agriculture are essential to stop the depletion of our soils and our biodiversity. Thus, increasing the research and applications of PGP of drought-resistant plants of the Mediterranean diet will fulfill the one health approach while applying the Farm to Fork Strategy that aims to accelerate the transition to a sustainable food system [89].

## 6. Conclusions

Mediterranean functional foods and biofortified items display nutrigenomic effects by modulating gene expression. The ability of these bioactive compounds to influence epigenetic mechanisms plays a crucial role in mitigating the risk of chronic diseases, such as cardiovascular ailments, cancer, and metabolic disorders. Recently, consumer interest in natural products has grown due to the increased awareness that a healthy lifestyle based on the consumption of high-quality products contributes to longevity. Thus, the need to underline the importance for consumers to take advantage of quality products began to grow, and a focus on all those molecules that can be extracted from plants, even from the less renowned, with specific climate standards was necessary.

Among the various bioactive molecules, those presented are part of a restricted pool, limited to drought-resistant edible plants from the Mediterranean region, that, in addition to presenting several beneficial biological effects, also have nutrigenomic activity, capable of modulating gene expression of those cells with which they come into contact, most often having beneficial purposes. 

Two reasons were behind the choice to explore these aspects in depth: the first due to the role that the Mediterranean diet holds within human lifestyles, and the second due to the need to make the most of those sources that can naturally cope with the increasing effects of climate change. A highlight on the microbiome of the presented species has even been proposed, as it is widely assumed that microorganisms play an increasingly pivotal role in soil quality and fertility and in contributing to plant resistance to biotic and abiotic stresses.

Although various application studies, especially in nutrition and medicine, are available, much remains undiscovered about these compounds, including their abundance and distribution. Advances in nutrigenetic, nutrigenomic, and nutrigenetic knowledge [90] will be necessary since they have recently achieved a prominent position in scientific and social interest. The evident correlation between the use of these compounds and their beneficial action against cancer, diabetes, and cardiovascular diseases will certainly stimulate new and more detailed studies, and hopefully, in the near future, more in-depth knowledge can be developed on what are the synergistic effects of microorganisms on the bioactive molecule composition of plants, to produce drugs, essential oils targeted to humans for their personal well-being. To gain more insights in those regards, coordinated and multidisciplinary research projects are necessary, needing public and private financial support to carry out high-throughput experiments, such as those based on omic technology, and mining their interconnected significance. The large number of multidisciplinary contributions present in the literature, also in the form of systematic reviews, represents a large reservoir of data that, however, does not allow specificity. The choice made in this review to limit the description to a few molecules and some plants has the precise objective of increasing specificity in order to give visibility to specific virtuous pathways with a high degree of correlation between their components, which can be candidates for high priority for future integrated research.

In conclusion, functional foods and biofortified products derived from Mediterranean foods play a pivotal role in promoting human health and preventing chronic diseases on one side while increasing the sustainability of our agricultural systems. Interestingly, drought tolerance and bioactive molecule production may be common factors in the specialized role of Mediterranean plant microbiota. The diverse array of bioactive compounds and enriched nutrients offer substantial health benefits, emphasizing the importance of integrating these dietary components into a balanced and nutritious diet. Understanding the nutrigenomic impact of these foods helps to develop targeted dietary strategies to promote human health, prevent disease, or, more interestingly, achieve a modulation effect on DNA damage resulting from exposure to genotoxic foods and drinks, a property already demonstrated for stilbenoids [91].

## Figures and Tables

**Table 1:** Microbiota of the four Mediterranean plant species presented in this review.

Mediterranean Plant	Family	Compartment	Most Abundant Taxa	References
*Origanum vulgare L.*	Lamiaceae	Seeds	*Pseudomonas; Bacillus;Pantoea*	[83,84]
		Stems	*Bacillus; Curtobacterium*	
		Leaves	*Arthrobacter; Bacillus*	
		Flowers	*Pantoea; Pseudomonas; Bacillus*	
*Capparis spinosa*	Capparaceae	Rhizosphere	*Pseudomonas; Agrobacterium; Sphingobacterium;*	[85]
*Thymus vulgaris L*.	Lamiaceae	Rhizosphere, Roots, Leaves	*Pseudomonas; Enterobacteriaceae*	[86]
*Opuntia ficus indica L. Mill*	Cactaceae	Endosphere, Rhizosphere	*Proteobacteria; Actinobacteria; Firmicutes; Cyanobacteria*	[87]

## Data Availability

The original contributions presented in the study are included in the article, further inquiries can be directed to the corresponding author.
